# Preliminary screening of biomarkers and drug candidates in a mouse model of β-thalassemia based on quasi-targeted metabolomics

**DOI:** 10.3389/fphys.2024.1452558

**Published:** 2024-08-23

**Authors:** Xianfeng Guo, Xuchao Zhang, Min Li, Yuanliang Peng, Zi Wang, Jing Liu

**Affiliations:** ^1^ Department of Hematology, The Second Xiangya Hospital, Molecular Biology Research Center, Center for Medical Genetics, School of Life Sciences, Hunan Province Key Laboratory of Basic and Applied Hematology, Central South University, Changsha, China; ^2^ Department of medical laboratory college, Changsha Medical University, Changsha, China

**Keywords:** β-thalassemia, quasi-targeted metabolomics biomarkers, drug candidates, machine learning algorithms, molecular docking

## Abstract

**Background:**

β-thalassemia (β-TH) is a hereditary hemolytic anemia that results in deficient hemoglobin (Hb) synthesis. It is characterized by ineffective erythropoiesis, anemia, splenomegaly, and systemic iron overload. Exploration new potential biomarkers and drug candidates is important to facilitate the prevention and treatment of β-TH.

**Methods:**

We applied quasi-targeted metabolomics between wild type (Wt) and heterozygous β-TH mice (Th3/+), a model of non-transfusion-dependent β-TH intermedia, in plasma and peripheral blood (PB) cells. Futher data was deeply mined by Kyoto Encyclopedia of Genomes (KEGG) and machine algorithms methods.

**Results:**

Using KEGG enrichment analysis, we found that taurine and hypotaurine metabolism disorders in plasma and alanine, aspartate and glutamate metabolism disorders in PB cells. After systematically anatomize the metabolites by machine algorithms, we confirmed that alpha-muricholic acid^UP^ and N-acetyl-DL-phenylalanine^UP^ in plasma and Dl-3-hydroxynorvaline^UP^, O-acetyl-L-serine^UP^, H-abu-OH^UP^, S-(Methyl) glutathione^UP^, sepiapterin^DOWN^, and imidazoleacetic acid^DOWN^ in PB cells play key roles in predicting the occurrence of β-TH. Furthermore, Sepiapterin, Imidazoleacetic acid, Methyl alpha-D-glucopyranoside and alpha-ketoglutaric acid have a good binding capacity to hemoglobin E through molecular docking and are considered to be potential drug candidates for β-TH.

**Conclusion:**

Those results may help in identify useful molecular targets in the diagnosis and treatment of β-TH and lays a strong foundation for further research.

## 1 Introduction

β-thalassemia (β-TH) is a hereditary hemolytic anemia that results in deficient hemoglobin (Hb) synthesis due to reduced or complete absence of synthesis of the β peptide chain of β-Hb (Taher et al., 2018; Thein, 2018). β-TH carriers comprise approximately 3% of the total global population (Jaing et al., 2021). It is estimated that about 300,000 to 500,000 children, the vast majority of whom are from developing countries, are born each year with severe hemoglobin abnormalities. Europe, Africa, the Mediterranean basin, the Middle East, continental India, Southeast Asia, and the Pacific Islands are severely affected. The prevalence of β-TH carriers in these regions ranges from 1% to 20%. Europe has the highest prevalence of about 35%, followed by Asia at 24%. Australia and South America have almost equal prevalence at 20% (Jaing et al., 2021).

The three provinces with the highest β-TH carriage rates in China are Guangxi (6.66%), Hainan (5.11%), and Guizhou (4.63%) (Shang et al., 2017). Guangdong, Yunnan, Hong Kong, Hunan, and Jiangxi also had high rates of carriage. According to the data of the China Thalassemia Blue Book (2020), there are about 30 million thalassemia gene carriers and about 300,000 patients with thalassemia major and intermedia in mainland China, and the number of affected people is increasing at a rate of approximately 10% per year.

There are several techniques for screening and diagnosing hemoglobin variants and thalassemia (Hartwell et al., 2005). Determining a patient’s genetic makeup and characteristics by complete blood count (CBC) is the most reliable method of diagnosing thalassemia (Birndorf et al., 1996). However, these methods do not provide insights into changes in metabolite patterns in biological material, which can provide valuable phenotypic information and mechanistically reveal the disease process and associated abnormal biochemical processes. Several studies have demonstrated that metabolic disorders are prevalent in patients with β-thalassemia (De Sanctis et al., 2013; De Sanctis et al., 2016), but to date, metabolic disorders remain unrecognized.

In the present study, we conducted quasi-targeted metabolomic analyses in β-TH and found metabolic disorders in plasma and peripheral blood (PB) cells. Analyzing the differential metabolites of Th3/+ mice in parallel with Wt mice led us to discover novel potential biomarkers and drug candidates for β-TH.

## 2 Materials and methods

### 2.1 Mice

Wild-type C57BL/6 (Wt) mice were obtained from the Hunan SJA. Laboratory Animal Co. Ltd. β-TH (Th3/+) mice with a C57BL/6 background were originally purchased from Jackson Laboratories. Male Th3/+ and female wild-type C57BL/6 mice were cross-bred to produce β-TH and wild-type littermates at Central South University Laboratory Animal Center.

### 2.2 Quasi-targeted metabolomic analysis

Using the eyeball removal method, we collected PB in EP tubes containing heparin (50 U/mL) and centrifuged it at 200 × g for 5 min at 4°C. The upper layer of fluid was plasma, and the lower layer of cells was PB cells. The plasma and PB cells were separated. PB cells (100 μL, about 1×10^9^–5×10^9^ cells) were ground separately with liquid nitrogen and resuspended in prechilled 80% methanol and 0.1% formic acid by vortexing. Samples were incubated on ice for 5 min and then centrifuged at 15,000 × g for 20 min at 4°C. Quasi-targeted metabolomics profiling was performed on the prepared samples using the XploreMET platform (Novogene, China) following previously reported procedures with minor modifications (Lin et al., 2023).

### 2.3 Biomarker screening

The random forest (RF) machine learning method was selected to construct the prediction model. Differential biomarkers in the top 15 positions that had a key role in the grouping were screened. Then, we performed Pearson’s correlation coefficient analysis of hemoglobin concentration and metabolite abundance in each mouse to further screen biomarkers by correlation coefficient (R^2^), *p*-values, and receiver operating characteristic curve.

### 2.4 Molecular docking

The crystal structure of the candidate protein targets of hemoglobin was downloaded from the RCSB Protein Data Bank (Th) and modified using the Autodock Tools 1.5.6 software. These targets include ligand and water removal, hydrogen addition, and amino acid optimization and patching. The files were saved in pdbqt format. Discovery Studio2019 Client was used to visualize the docking results.

### 2.5 Statistical analysis

The statistical analysis of each plot is described above or in the corresponding figure legend. All grouped data values are presented as the mean ± SD. *p*-values were calculated using Student’s t-test or Kruskal–Wallis ANOVA with GraphPad Prism software.

## 3 Results

### 3.1 Basic characteristics of Th3/+ mice

Female Wt C57BL/6 mice and Male Th3/+ mice were crossed to produce β-TH and Wt littermates. Five male Th3/+ mice and five male Wt mice were selected by genotype. Routine blood tests revealed that Hb, red blood cells (RBC), hematocrit (HCT), mean corpuscular volume (MCV), mean corpuscular hemoglobin (MCH), and mean corpuscular hemoglobin concentration (MCHC) were significantly lower, while reticulocyte percentage (ret%) was significantly higher in Th3/+ mice (Table 1). The results illustrate that the genotypic identification results were consistent with the phenotypic results, which lays the foundation for the accuracy of the subsequent experimental results.

**TABLE 1:** Mouse red blood cell parameters.

Treatment group	Hb (g/L), mean (SD)	RBC (10^12^/L), mean (SD)	Ret (%), mean (SD)	HCT (%), mean (SD)	MCHC (g/L), mean (SD)	MCV (fL), mean (SD)
Wt	138.6 (3.4)	8.9 (0.2)	3.14 (0.7^)^	39.9 (1.1)	347 (4.6)	44.7 (0.3)
Th3/+	79.6 (4.8)^***^	7.8 (0.5)^**^	31.84 (3.4)^***^	28.8 (1.6)^***^	276.6 (5.1)^***^	37.1 (1.2)^***^

Note: Hemoglobin, RBC, Ret%, HCT, MCHC, and MCV in the Wt and Th3/+ mice. N = 5 mice per group. Hb: hemoglobin, RBC: red blood cells, Ret: reticulocyte, HCT: hematocrit, MCHC: mean corpuscular hemoglobin concentration, MCV: mean corpuscular volume. Th3/+: β-thalassemic, Wt: wild type. ***p < 0.001 and **p < 0.01.

For preliminary screening of biomarkers and drug candidates in Th3/+ mice, we selected PB cells and plasma for metabolomic analysis. The combined analysis of plasma and PB cells could reflect the metabolic status inside and outside the cells. In addition, compared with erythrocytes, PB cells have a wider detection range, which can provide more options for screening markers and drug candidates.

### 3.2 Analysis of the metabolome data

#### 3.2.1 QC analysis

**FIGURE 1:**
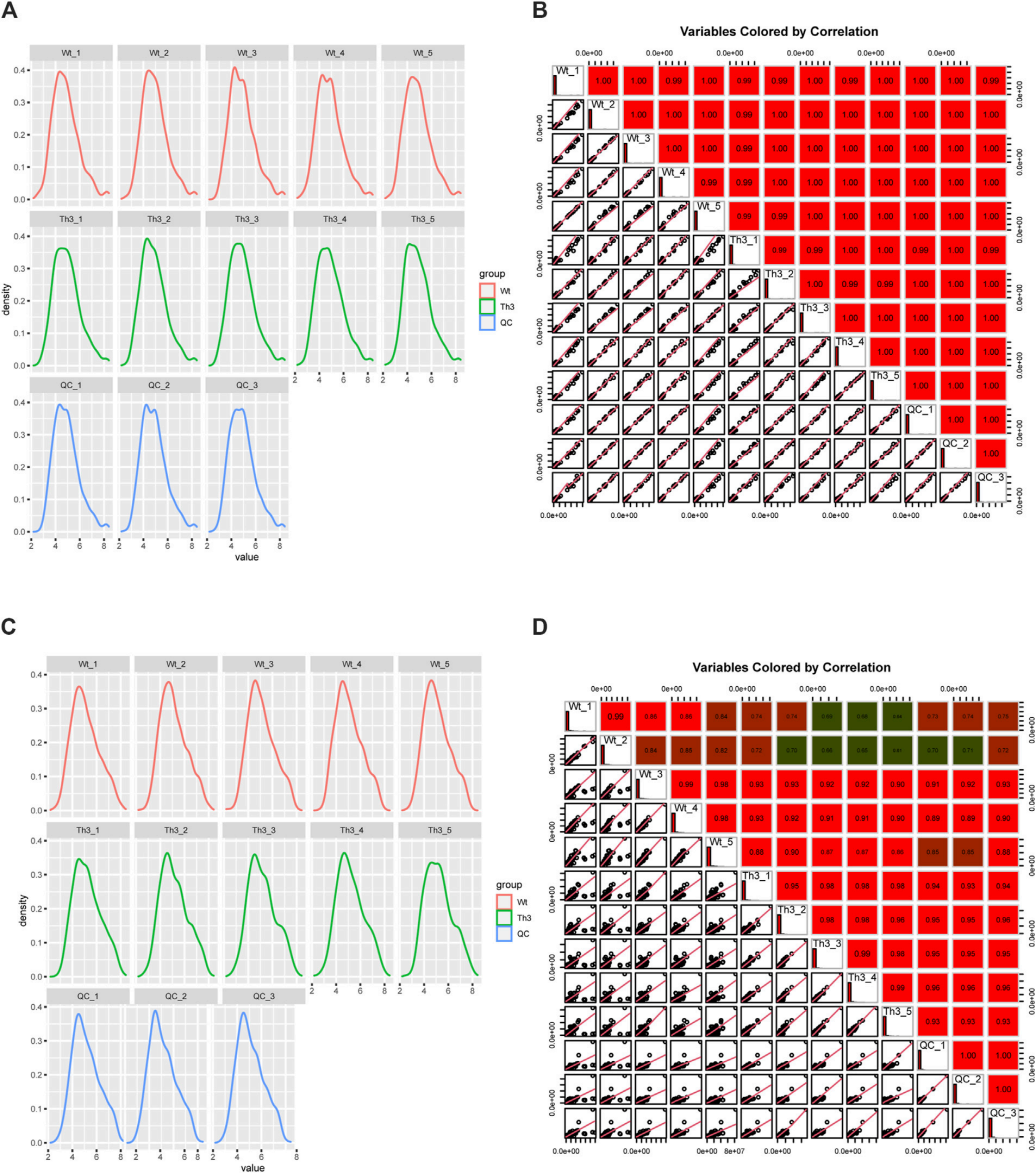
QC analysis of the metabolome data. **(A, B)** were plasma, and **(C, D)** were peripheral blood cells (PB cells).

The kernel density estimation plot (Kdeplot) reveals that the plasma distribution of metabolites is good (Figure 1A). The Pearson correlation coefficients of QC samples calculated by the relative quantitative values of metabolites are between 0.99 and 1.00 (Figure 1B). The Kdeplot of PB cell distribution of metabolites is also good (Figure 1C), while the Pearson correlation coefficients of QC samples were between 0.61 and 1.00 (Figure 1D). The above data indicate that the quality of these data is high, laying the foundation for subsequent research.

#### 3.2.2 Differential metabolite analysis of PB cells and plasma between Th3/+ mice and Wt mice

**FIGURE 2:**
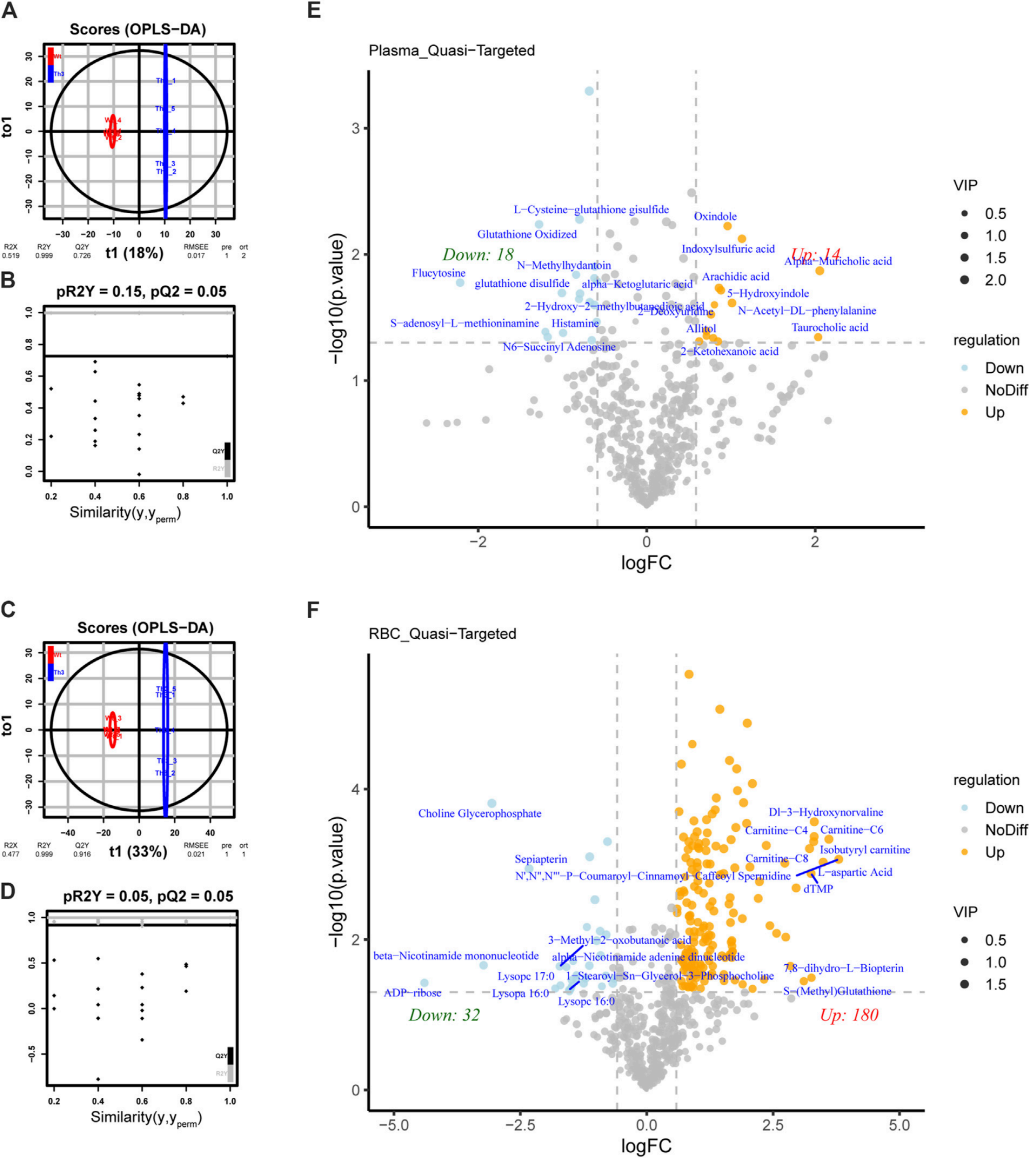
Differential metabolite analysis of PB cells and plasma between Th3/+ mice and Wt mice. **(A)** Orthogonal partial least squares discriminant analysis (OPLS-DA) of plasma. **(B)** OPLS-DA score plots from the Wt mice and Th3/+ mice in plasma (pR2 = 0.15, pQ2 = 0.05). **(C)** OPLS-DA of PB cells. **(D)** OPLS-DA score plots from the Wt mice and Th3/+ mice in PB cells (pR2 = 0.05, pQ2 = 0.05). Volcano plot of differentially abundant metabolites in plasma **(E)** and PB cells **(F)**. The horizontal coordinate indicates the variation in the differential multiplicity of metabolites in different subgroups (log2FC), and the vertical coordinate indicates the level of differential significance (−log10(*p*-value)). Each point in the volcano plot represents a metabolite. Significantly upregulated metabolites are represented by yellow dots, significantly downregulated metabolites are represented by blue dots, and the size of the dots represents the VIP value.

We applied OPLS-DA to plasma (Figures 2A,B) and PB cells (Figures 2C,D) for statistical analysis. We obtained model evaluation parameters R2 and Q2 by 7-fold cross-validation, with the results suggesting that the models were good, and we could proceed to the next analysis step. After analyzing the targeted metabolic data, we found that the difference in plasma metabolites between groups was obvious among the 649 metabolites tested (Figure 2E). In PB cells, the differential metabolite expressions between groups were also obvious among the 752 metabolites tested (Figure 2F).

Thirty-two metabolites in plasma were differentially expressed between Th3/+ mice and Wt mice, of which 18 metabolites showed an upward trend and 14 metabolites showed a downward trend. In PB cells, 212 metabolites were differentially expressed, of which 180 metabolites showed an upward trend and 32 metabolites showed a downward trend. The differences among groups were further clarified.

#### 3.2.3 Hierarchical clustering analysis of the differential metabolites

Compared to Wt mice, the main upward-trending metabolites were concentrated in bile acids, while the main downward-trending metabolites were concentrated in amino acids in plasma. In PB cells, the main upward-trending metabolites were concentrated in amino acids, while the main downward-trending metabolites were concentrated in phospholipids.

**FIGURE 3:**
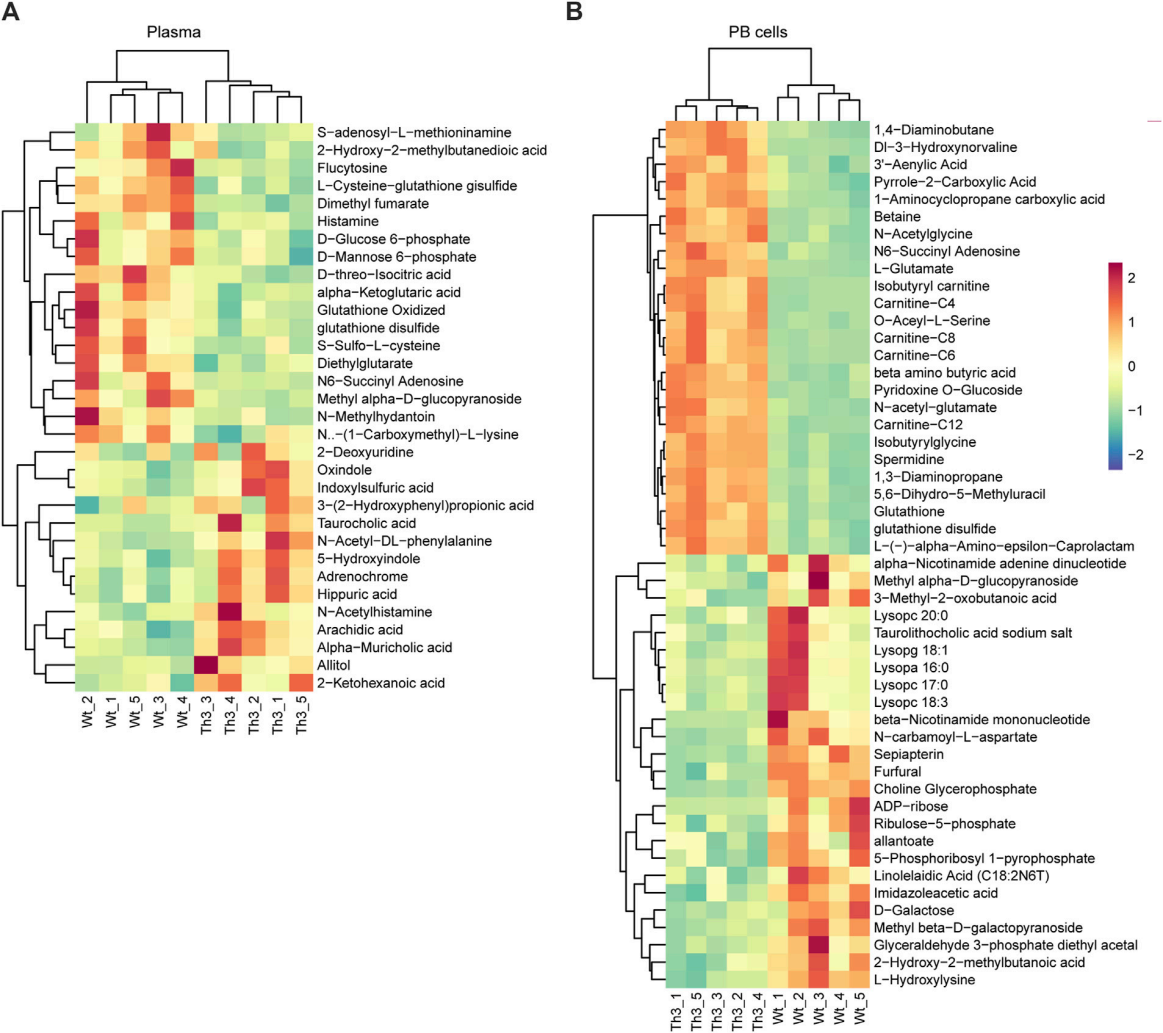
Hierarchical clustering analysis of the differential metabolites: heat map analysis of plasma **(A)** and PB cells **(B)**.

Hierarchical clustering showed a clearer picture of the detailed clustering of the top 25 differential metabolites. In plasma, the top upregulated metabolites were 2-deoxyuridine, oxindole, indoxylsulfuric acid, and 3-(2-hydroxyphenyl) propionic acid, while the top downregulated metabolites were S-adenosyl-L-methioninamine, 2-hydroxy-2-methylbutanedioic acid, flucytosine, and L-cysteine-glutathione disulfide (Figure 3A). In PB cells, the top upregulated metabolites were 1,4-diaminobutane, DI-3-hydroxynorvaline, adenylic acid, and pyrrole-2-carboxylic acid, while the top downregulated metabolites were alpha-nicotinamide adenine dinucleotide, methyl alpha-D-glucopyranoside, 3-methyl-2-oxobutanoic acid, and lysoPC 20:0 (Figure 3B).

Although the number of results is substantial, there are still differences between mice of the same type, especially in plasma. Therefore, we need other methods to better analyze metabolites.

#### 3.2.4 KEGG enrichment analysis in different groups

**FIGURE 4:**
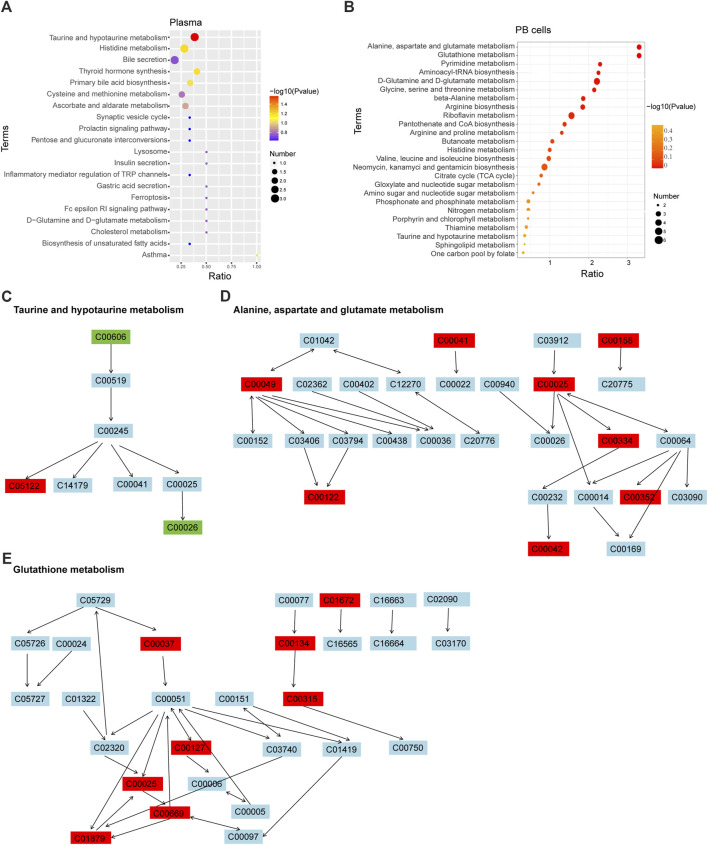
KEGG enrichment analysis in different groups. Bubble diagram of KEGG enrichment of plasma **(A)** and PB cells **(B)**. The color of the dot represents the *p*-value, and the size of the dot represents the number of differential metabolites in the corresponding pathway. Schematic diagram of metabolic pathways of taurine and hypotaurine metabolism **(C)**; alanine, aspartate, and glutamate metabolism **(D)**; and glutathione metabolism **(E)**.

KEGG enrichment analysis of the differential metabolites showed that plasma of Th3/+ mice and Wt groups were mainly enriched in taurine and hypotaurine metabolism (*p* = 0.025) (Figure 4A), while PB cells were mainly enriched in alanine, aspartate, and glutamate metabolism (*p* < 0.001) and glutathione metabolism (*p* < 0.001) (Figure 4B). In taurine and hypotaurine metabolisms, the differential metabolites were 3-sulfino-L-alanine^DOWN^, taurocholate^UP^, and alpha-ketoglutaric acid^DOWN^(Figure 4C). In alanine, aspartate, and glutamate metabolism, the differential metabolites were L-alanine^UP^, citrate^UP^, L-aspartate^UP^, L-glutamate^UP^, 4-aminobutanoate^UP^, fumarate^UP^, D-glucosamine 6-phosphate^UP^, and succinate^UP^(Figure 4D). In glutathione metabolism, the differential metabolites were cadaverine^UP^, glycine^UP^, putrescine^UP^, spermidine^UP^, glutathione disulfide^UP^ (GSH), L-glutamate^UP^, gamma-L-glutamyl-L-cysteine^UP^, and 5-oxoproline^UP^(Figure 4E).

In addition to these top-ranked metabolic pathways, pyrimidine metabolism, aminoacyl-tRNA biosynthesis, and D-glutamine and D-glutamate metabolism were strengthened in the PB cells of Th3/+ mice, with the levels of related metabolites significantly higher than those in normal controls. From the metabolic pathways, we can speculate that the large number of upregulated metabolites in Th3/+ mice is closely related to the enhancement of metabolism. These increased metabolites also offer the possibility of searching for biomarkers associated with anemia in Th3/+ mice.

### 3.3 Biomarker prediction of β-TH

#### 3.3.1 Machine learning algorithms for β-TH biomarker prediction

**FIGURE 5:**
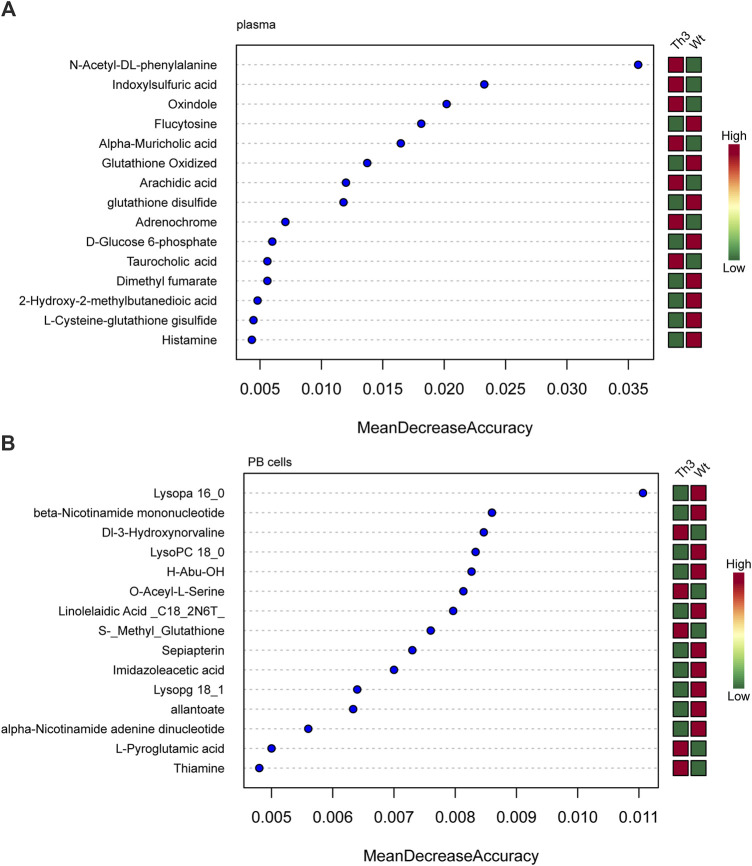
Machine learning algorithms for β-TH biomarker prediction: random forest (RF) analysis of plasma **(A)** and PB cells **(B)**.

The metabolites with variable importance in projection (VIP) > 1.0 and *p*-value < 0.05 were chosen, and comparisons were made among each group using RF to screen the top 15 metabolites. After combined analysis of the machine learning algorithms, the representative difference products between Th3/+ mice and Wt mice in plasma were N-acetyl- DL-phenylalanine, indoxylsulfuric acid, oxindole, flucytosine, alpha-muricholic acid, oxidized glutathione, arachidic acid, and glutathione disulfide (mean decrease accuracy>0.01, Figure 5A).

The combined analysis of the machine learning algorithms revealed that the representative differential products in the PB cells of Th3/+ mice and Wt mice were lysoPA 16:0, beta-nicotinamide mononucleotide, DI-3-hydroxynorvaline, LysoPC 18:0, H-abu-oh, O-acetyl-L-serine, linolelaidic acid, S-methyl glutathione, sepiapterin, and imidazoleacetic acid (mean decrease accuracy > 0.007, Figure 5B).

#### 3.3.2 Further screening of predicted biomarkers

**FIGURE 6:**
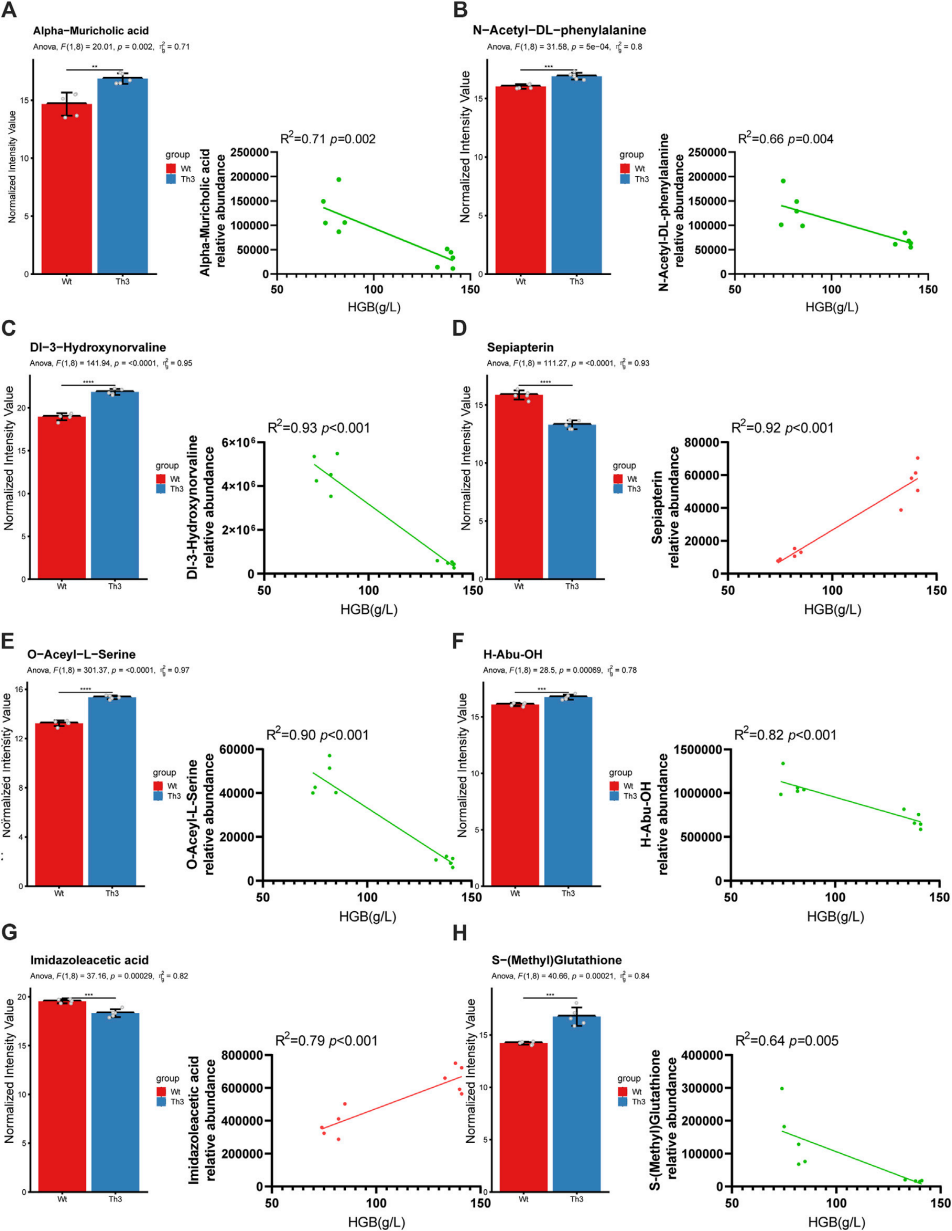
Further screening of predicted biomarkers. Relative abundance and correlation coefficient of alpha-muricholic acid **(A)**, N-acetyl-DL-phenylalanine **(B)**, Dl-3-hydroxynorvaline **(C)**, sepiapterin **(D)**, O-acetyl-L-serine **(E)**, H-abu-OH **(F)**, imidazoleacetic acid **(G)**, and S-(methyl) glutathione **(H)**. Comparisons between the two groups were evaluated with a 2-tailed t-test, *p < 0.05; **p < 0.01; ***p < 0.001.

We performed Pearson’s correlation coefficient analysis of RF-screened biomarkers with mice Hb and found that in plasma, the alpha-muricholic acid (R = 0.84, *p* = 0.002) and N-acetyl-DL-phenylalanine (R = 0.81, *p* = 0.004) were highly correlated with hemoglobin (Figures 6A,B). In PB cells, Dl-3-hydroxynorvaline (R = 0.96, *p <* 0.001), sepiapterin (R = 0.95, *p <* 0.001), O-acetyl-L-serine (R = 0.94, *p <* 0.001), H-abu-OH (R = 0.90, *p <* 0.001), imidazoleacetic acid (R = 0.88, *p <* 0.001) and S-(methyl) glutathione (R = 0.8, *p* = 0.005) were highly correlated with mice hemoglobin (Figures 6C–H). In addition, the area under the curve (AUC) values of the above metabolites were estimated, and the results showed that all metabolites had an AUC value of 1 (Table 2).

**TABLE 2:** Information of predicted biomarkers.

Groups	Metabolites	Formula	Class	Trend	ROC
Plasma	Alpha-muricholic acid	C_24_H_40_O_5_	Bile acids	Up	1.00
	N-acetyl-DL -phenylalanine	C_11_H_13_NO_3_	Amino acid and its derivatives	Up	1.00
PB cells	DI-3-hydroxynorvaline	C_5_H_11_NO_3_	Amino acid and its derivatives	Up	1.00
	Sepiapterin	C_9_H_11_N_5_O_3_	Organoheterocyclic compounds	Down	1.00
	O-acetyl-L-serine	C_5_H_9_NO_4_	Amino acid and its derivatives	Up	1.00
	H-Abu-OH	C_4_H_9_NO_2_	Amino acid and its derivatives	Up	1.00
	Imidazoleacetic acid	C_5_H_6_N_2_O_2_	Organic acid and its derivatives	Down	1.00
	S-(methyl) glutathione	C_11_H_19_N_3_O_6_S	Amino acid and its derivatives	Up	1.00

From the above results, we found that the screened serum metabolic markers alpha-muricholic acid and N-acetyl-DL-phenylalanine were elevated. In PB cells, Dl-3-hydroxynorvaline, O-acetyl-L-serine, H-abu-OH, and S-(methyl) glutathione were elevated while sepiapterin and imidazoleacetic acid were decreased. We speculate that alpha-muricholic acid^UP^, N-acetyl-DL-phenylalanine^UP^, Dl-3-hydroxynorvaline^UP^, O-acetyl-L-serine^UP^, H-abu-OH^UP^, S-(methyl) glutathione^UP^, sepiapterin^DOWN^, and imidazoleacetic acid^DOWN^ play key roles in predicting the occurrence of β-TH.

### 3.4 Drug candidate prediction of β-TH

A combined analysis of the differential metabolites in plasma and PB cells revealed 21 common differential metabolites, of which eight were co-upregulated, and three were co-downregulated. The three co-downregulated metabolites were methyl alpha-D-glucopyranoside, alpha-ketoglutaric acid, and 2-hydroxy-2-methylbutanoic acid. Together with sepiapterin and imidazoleacetic acid, the above five metabolites were selected as drug candidates for β-TH.

**FIGURE 7:**
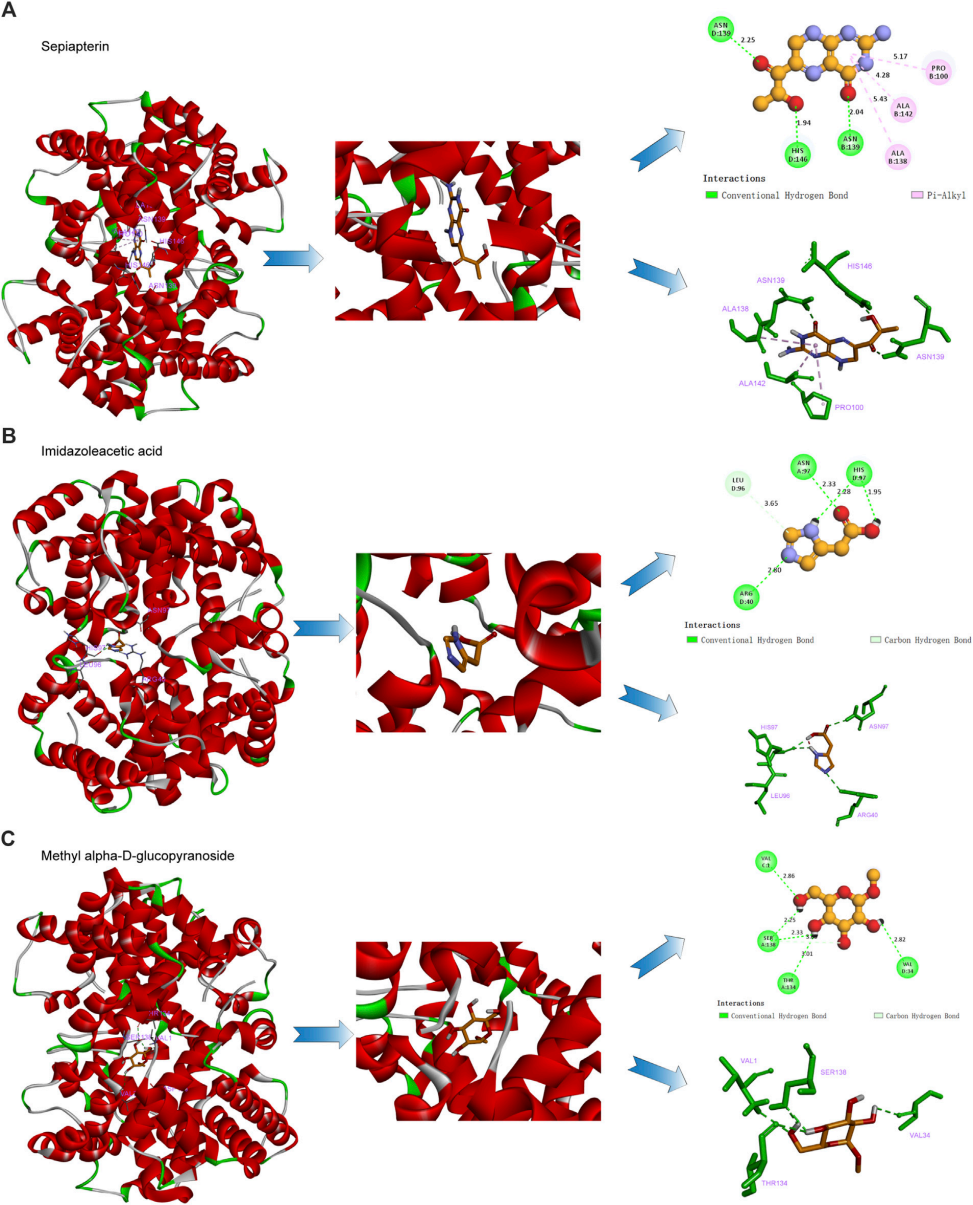
β-TH drug candidate prediction. Molecular docking analysis of the interaction of the hemoglobin E with sepiapterin **(A)**, imidazoleacetic acid **(B)**, and methyl alpha-D-glucopyranoside **(C)**.

The interaction of hemoglobin E (1NQP) isolated from blood samples of β-TH predictive candidate targets was validated by molecular docking. The 2D and 3D structures of the ligands are shown in Figure 7. The estimated free energy of binding is summarized in Table 3. Electrostatic and van der Waals forces are the main forces between ligand and target proteins. The binding energies of the ligands and receptors of sepiapterin, imidazoleacetic acid, methyl alpha-D-glucopyranoside, and alpha-ketoglutaric acid were less than −5.366 kcal/mol with 1NQP, indicating good binding stability between the above compounds and the 1NQP target.

**TABLE 3:** Molecular docking energy results.

Protein pdb ID	Compound	Minimum docking energy (kcal/mol)
1NQP	Sepiapterin	−6.675
	Imidazoleacetic acid	−5.084
	Methyl alpha-D-glucopyranoside	−5.032
	Alpha-ketoglutaric acid	−5.366
	2-Hydroxy-2-methylbutanedioic acid	−4.882

## 4 Discussion

Metabolites serve as direct signatures of biochemical activity and provide a readout of the cell state. Metabolomic analysis has identified 40 serum metabolites that are significantly differently expressed between β-TH patients and healthy controls, and metabolic pathway analysis revealed multiple alterations (Musharraf et al., 2017). A metabolomic study in the serum of 40 β-thalassemia patients before and after administration of hydroxyurea (HU) revealed that the levels of 25 metabolites, which were altered before the patients received HU therapy, started to revert toward the levels of the healthy group after HU treatment. As HU shifts body metabolism toward normal, this study suggested that HU is a good treatment option and can ameliorate disease complications (Iqbal et al., 2018). Most previous studies were single-sample studies. We designed plasma and PB cells for joint metabolomic analysis. In addition, we selected quasi-targeted metabolomics methods, which are a new technology integrating sequencing breadth and accuracy (Wang et al., 2022; Wu et al., 2023; Peng et al., 2024) and discovered many new metabolic biomarkers and drug candidate metabolites.

Using KEGG analysis, we found disturbed metabolism of alanine, aspartate, and glutamate in PB cells with nine metabolite changes, most of which were related to energy metabolism, such as citrate, fumarate, and succinate. Matte A et al. demonstrated a significant reduction of ATP in the bone marrow and splenocytes of Th3/+ mice (Matte et al., 2021). The reduction of ATP in erythrocytes affects the stability of the cell membrane and leads to hemolysis. This is the first time we have focused on metabolite changes in energy supply, prompting us to explore the new pathogenesis of β-TH. Another metabolism disorder in PB cells is glutathione metabolism, and GSH plays a core role. GSH is essential for cellular redox homeostasis (Seo et al., 2004), and the glutathione redox system is the first line of defense against oxidative stress in thalassemia patients (Kalpravidh et al., 2013). In terms of pathogenesis, loss of HBB gene function in human erythroid progenitor cells leads to increased ROS generation and oxidative stress, causing increased apoptosis. Therefore, by metabolomics assay, we speculate that the increase of ROS in Th3/+ mice was related to glutathione metabolism.

We identified eight potential metabolic markers for the diagnosis of thalassemia, most of which were derived from amino acids and derivatives (62.5%). A study of the amino acid metabolism of β-TH major patients in the United Arab Emirates found that glutamate, serine, and proline were significantly higher, which was consistent with our results (Abdulrazzaq et al., 2005). According to KEGG analysis, amino acids represented by glycine were significantly increased in the glutathione metabolism disorder in PB cells. In erythroblasts, glycine uptake via the glycine carrier system, GlyT1, is a rate determiner of heme biosynthesis and bioavailability (Eulenburg et al., 2005; Wolosker, 2007), and an oral selective inhibitor of GlyT1 was developed to treat negative symptoms of patients with schizophrenia (Black et al., 2009; Roberts et al., 2010; Umbricht et al., 2014; Winter et al., 2016) and also had a good treatment effect in a mouse model of β-TH (Matte et al., 2019). Developing drugs that reduce these upregulated metabolites is a new approach to drug discovery.

Hemoglobin E [HbE; Glu26(B8) → Lys], a result of splice site mutation (GAG → AAG) in exon 1 of the β-globin gene (Orkin et al., 1982; Shirohzu et al., 2000), is prevalent in Southeast Asia (Weatherall and Clegg, 1996) and the most common hemoglobin variant. HbE combined with β-thalassemia leads to E/β-thalassemia, with severe clinical consequences. E/β-thalassemics and homozygous HbE showed the instability of HbE in a higher temperature; in particular, any change that reduces the contacts between the subunits that compose the hemoglobin would generate its instability (Rees et al., 1998; Sen et al., 2004). Using molecular docking, we found that sepiapterin, imidazoleacetic acid, methyl alpha-D-glucopyranoside, and alpha-ketoglutaric acid have good binding forces, suggesting that those metabolites might increase HbE stability by increasing contacts between the subunits.

Among them, sepiapterin was predicted to be the first candidate metabolic drug. Tetrahydrobiopterin plays an important role in functional and metabolic cellular homeostasis, with additional effects on proliferation (Tanaka et al., 1989; Anastasiadis et al., 1997), immune responsiveness (Huber et al., 1984; Fukuda et al., 1985), and neuronal activity (Mataga et al., 1991; Koshimura et al., 1995; Koshimura et al., 2000). Sepiapterin, an analog of tetrahydrobiopterin, has shown good therapeutic effects in many diseases (Tarpey, 2002). Sepiapterin has been reported to diminish eNOS-derived superoxide in human vascular segments (Guzik et al., 2002). Spontaneously diabetic BB rats have diminished GTP-cyclohydrolase 1 activity and decreased BH4 levels, while sepiapterin treatment normalized BH4 levels for 48 h and increased NO production from endothelial cells isolated from diabetic BB rats (Meininger et al., 2000). Vasquez-Vivar et al. found a 6 h influence of sepiapterin incubation on vascular BH4 levels and endothelial function in vessels isolated from rabbits fed a high-cholesterol diet (Vasquez-Vivar et al., 2002). From the above results, we speculated that sepiapterin might be beneficial to β-TH and need further investigation.

In this study, we chose plasma and PB cells for metabolomic analysis. However, due to the large differences in circulating cells in Th3/+ mice compared to WT mice, it is necessary to isolate these cells and analyze individual cell populations so that the metabolic status of each cell population can be more accurately reflected. In addition, as a preliminary study for screening biomarkers and drug candidates, these results must be confirmed by further analysis using human cells.

## 5 Conclusion

In the present study, we conducted quasi-targeted metabolomics analyses and found dysfunctional metabolism in Th3/+ mice plasma and PB cells. The complex pathogenesis of Th3/+ remains unclear. This study systematically analyzed the metabolites further preliminarily confirming that alpha-muricholic acid^UP^ and N-acetyl-DL-phenylalanine^UP^ in plasma and Dl-3-hydroxynorvaline^UP^, O-acetyl-L-serine^UP^, H-abu-OH^UP^, S-(methyl) glutathione^UP^, sepiapterin^DOWN^, and imidazoleacetic acid^DOWN^ in PB cells play key roles in predicting the occurrence of β-TH. Furthermore, sepiapterin, imidazoleacetic acid, methyl alpha-D-glucopyranoside, and alpha-ketoglutaric acid are drug candidates with a good binding capacity to hemoglobin E, laying a strong foundation for further investigation. Our findings may help identify useful molecular targets in the diagnosis and treatment of β-TH. However, owing to the complexity of metabolomics data, not all metabolites were systematically analyzed in this study. In subsequent studies, specimens from β-TH patients will be collected for targeted metabolomics analysis, and the above initial screening results will be validated in human cells.

## Data Availability

The original contributions presented in the study are included in the article/Supplementary Material; further inquiries can be directed to the corresponding author.
